# Supplementary material for: Equilibrium Adsorption of Organic Compounds (Nitrobenzene Derivative, Synthetic Pesticide, Dye, and Surfactant) on Activated Carbon from Single- and Multi-Component Systems
Source: Molecules. 2024 Dec 29;30(1):88. doi: 10.3390/molecules30010088 (PMC11721098; doi:10.3390/molecules30010088)
Supplement: Supplementary file 1 [file molecules-30-00088-s001.zip › molecules-3332904-supplementary.pdf]

SUPPORTING INFORMATION

Equilibrium adsorption of organic compounds (nitrobenzene derivative, synthetic pesticide, dye, and surfactant) on activated carbon from single- and multi-component systems

Magdalena Blachnio [1,*], Malgorzata Zienkiewicz-Strzalka [1], Anna Derylo-Marczewska [1]

Department of Physical Chemistry, Institute of Chemical Sciences, Faculty of Chemistry, Maria Curie-Sklodowska University, M. Curie-Sklodowska Sq. 3, 20-031 Lublin, Poland; malgorzata.zienkiewicz-strzalka@mail.umcs.pl (M.Z.-S.); anna.derylo-marczewska@mail.umcs.pl (A.D.-M.)

Table S1. Physicochemical properties of the studied pollutants.

Adsorbate	M [g/mol]	pK_a	Solubility [g/L]	Van der Waals volume [Å^3]	Maximal projection area [Å^2]
4-NA	138.12	1.00	0.57	115.97	47.44
4-CPA	186.59	3.14	0.96	149.71	56.50
AR 88	400.38	11.06	1.50	307.60	114.27
U	376.28	2.2; 4.4; 6.7	10	271.34	86.84

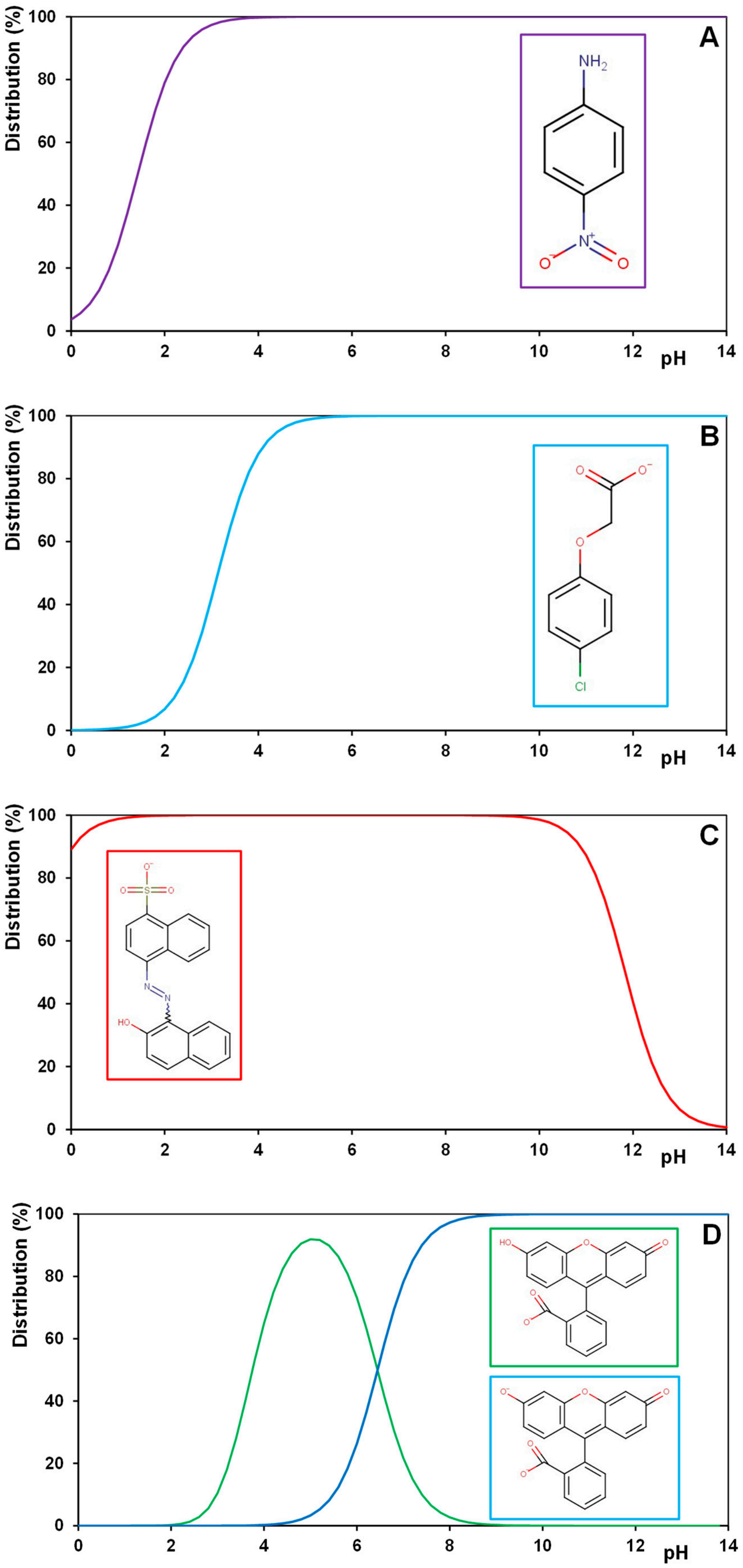

Figure S1. Percentage of molecular forms of 4-NA (**A**), 4-CPA (**B**), AR 88 (**C**), and U (**D**) as a function of pH solution.

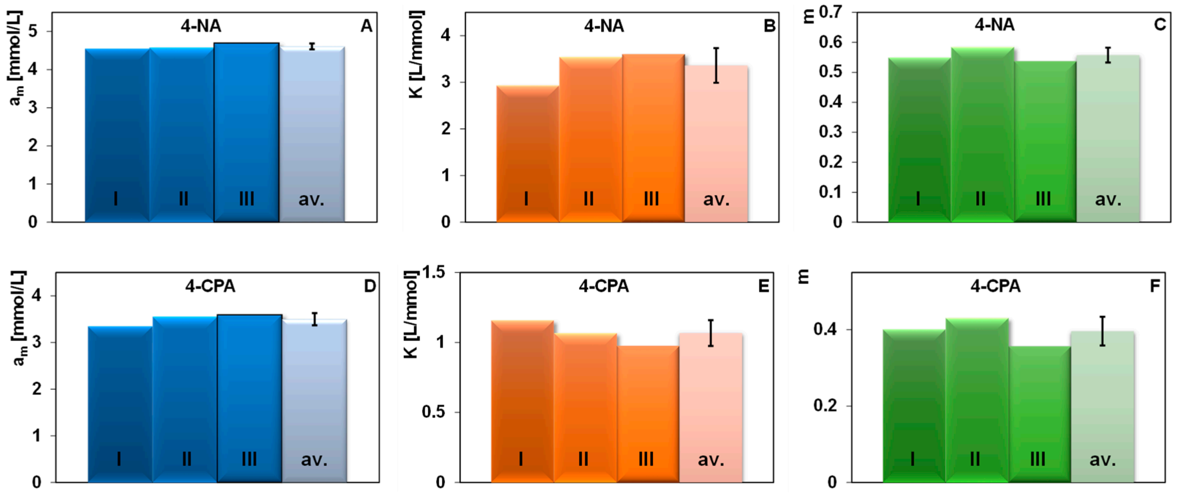

Figure S2. Graphical presentation of the values of Generalized Freundlich equation parameters a_m, m and K and their uncertainties, for independent series (I–III) of adsorption measurements of 4-NA (A, B, C) and 4-CPA (D, E, F) on the activated carbon RIAA.
